# Development of a risk prediction model for gastrointestinal adverse events associated with semaglutide administration in patients with type 2 diabetes mellitus

**DOI:** 10.3389/fendo.2025.1684395

**Published:** 2025-11-03

**Authors:** Deyong Yue, Xuesheng Hua, Ling Zhu, Jun Wang, Lihua Gu, Zhengxia Yuan, Wenle Jian, Yirong Chen, Guoliang Meng

**Affiliations:** ^1^ Chongming Hospital Affiliated to Shanghai University of Medicine and Health Sciences, Chongming, Shanghai, China; ^2^ Department of Pharmacology, School of Pharmacy, Nantong University, Nantong, Jiangsu, China; ^3^ Nantong University Xinglin College, Nantong, Jiangsu, China; ^4^ General Surgery Department of Wuxi No.8 People’s Hospital, Wuxi, Jiangsu, China

**Keywords:** semaglutide, type 2 diabetes mellitus, gastrointestinal adverse events, risk prediction model, alcohol, α-glucosidase inhibitors

## Abstract

**Objective:**

This study aims to identify the risk factors associated with gastrointestinal adverse events induced by semaglutide in patients with type 2 diabetes mellitus (T2DM) and to develop a predictive risk model.

**Methods:**

A total of 215 patients with T2DM admitted to our hospital between June 2022 and December 2024 were enrolled. Participants were divided into two groups based on the presence (*n* = 41) or absence (*n* = 174) of gastrointestinal adverse events associated with semaglutide use. Univariate and multivariate logistic regression analyses were performed to identify significant risk factors for these adverse events. Subsequently, a nomogram was developed using R software to predict the likelihood of gastrointestinal adverse events in this patient population. The predictive performance of the nomogram was assessed using receiver operating characteristic (ROC) curves, calibration plots, and the Hosmer–Lemeshow goodness-of-fit test.

**Results:**

In a cohort of 215 patients with T2DM, 41 individuals (19.07%) experienced gastrointestinal adverse events attributed to semaglutide administration. Logistic regression analysis identified concomitant gastrointestinal disorders [95% confidence interval (CI): 2.074–9.808, *P* < 0.001], alcohol consumption (95% CI: 1.304–6.633, *P* = 0.009), and concurrent use of α-glucosidase inhibitors (95% CI: 1.368–6.460, *P* = 0.006) as independent risk factors for semaglutide-induced gastrointestinal adverse events in this population. The predictive model demonstrated an area under the ROC curve of 0.763 (95% CI: 0.691–0.836). Calibration assessment revealed a slope approximating unity, and the Hosmer–Lemeshow goodness-of-fit test indicated an adequate model fit (χ² = 5.633, *P* = 0.228).

**Conclusion:**

Patients with T2DM exhibit a significant incidence of gastrointestinal adverse events associated with semaglutide use. The presence of gastrointestinal comorbidities, alcohol consumption, and α-glucosidase inhibitor therapy are independent risk factors. The developed nomogram effectively predicts the likelihood of semaglutide-related gastrointestinal adverse events in this patient population.

## 1 Introduction

Type 2 diabetes mellitus (T2DM) is a chronic metabolic disorder and represents the predominant form of diabetes (1–3). Persistent glycemic instability associated with T2DM leads to damage in various target organs, resulting in complications that diminish patients’ quality of life and adversely affect clinical outcomes (4–6). Pharmacological interventions constitute a critical component of T2DM management (7). Semaglutide, a glucagon-like peptide-1 (GLP-1) receptor agonist, has received regulatory approval for the treatment of T2DM (8). Beyond its primary glucose-lowering effect, semaglutide offers additional benefits, including weight reduction and cardioprotective properties (9–11). The glucose-lowering mechanism of semaglutide involves mimicking the activation of the GLP-1 receptor by endogenous GLP-1, thereby modulating insulin and glucagon secretion in a glucose-dependent manner (12). Semaglutide also exerts beneficial effects by modulating m6A modifications, thereby mitigating pancreatic beta cell dysfunction and influencing the composition of the gut microbiome (13). Additionally, semaglutide has demonstrated cardioprotective effects in the context of diabetic heart failure by mitigating cardiac inflammation via the Sirtuin 3-dependent Raf kinase inhibitor protein (RKIP) signaling pathway (14). Despite these therapeutic benefits, a substantial proportion of patients discontinue semaglutide within one year of initiation, primarily due to adverse effects, financial constraints, or diminished efficacy.

Previous studies have documented that adverse events related to the gastrointestinal system, induced by GLP-1 receptor agonist administration, are notably prevalent, with nausea and abdominal pain being the most frequently reported symptoms (15, 16). However, there is a paucity of data regarding gastrointestinal adverse events associated with semaglutide use in patients with T2DM in both domestic and international literature. Column-line diagrams, also known as nomograms, offer a more practical and user-friendly approach for visualizing predictive models (17, 18). Among the various forms of column charts, the line segment static column chart is the most widely used; it presents each predictor individually, assigns a specific score, and enables the calculation of predicted probabilities by summing the scores of all predictors.

Accordingly, this study aimed to identify the risk factors contributing to gastrointestinal adverse events related to semaglutide use in patients with T2DM and to develop a risk prediction model represented through a column-line diagram.

## 2 Methods

### 2.1 Study participants

Between June 2022 and December 2024, a total of 215 patients diagnosed with T2DM were recruited from Chongming Hospital affiliated to Shanghai University of Medicine and Health Sciences. The inclusion criteria were as follows: a confirmed diagnosis of T2DM (19), age over 18 years, initiation of semaglutide treatment for the first time, and provision of written informed consent. Exclusion criteria included presence of severe psychiatric disorders, diagnosis of malignant tumors, and pregnancy or lactation.

### 2.2 Demographic and clinical characteristics

The variables assessed included gender, age, body weight, duration of T2DM, glycosylated hemoglobin (HbA1c) levels, body mass index (BMI), liver enzymes including alanine aminotransferase (ALT) and aspartate aminotransferase (AST), and lipid profile components such as total cholesterol (TCHO), triglycerides (TG), low-density lipoprotein cholesterol (LDL-C), and high-density lipoprotein cholesterol (HDL-C). Blood pressure measurements comprised systolic blood pressure (SBP) and diastolic blood pressure (DBP). Other measured parameters comprised the presence of gastrointestinal diseases, alcohol consumption (defined as regular intake of any alcoholic beverage for more than six months), smoking status, and concurrent use of insulin and various antidiabetic medications, such as the number of oral hypoglycemic agents, metformin, sulfonylureas, α-glucosidase inhibitors, thiazolidinediones, sodium-glucose cotransporter 2 (SGLT2) inhibitors, and dipeptidyl peptidase 4 (DPP-4) inhibitors. Additionally, renal function status was evaluated.

### 2.3 Gastrointestinal adverse events

In this study, 215 patients with T2DM were divided into two groups based on the presence or absence of gastrointestinal adverse events associated with semaglutide administration. Follow-up was primarily conducted through review of patients’ electronic medical records, data from routine outpatient examinations, and telephone follow-ups to monitor and document the occurrence of adverse events. The identification of gastrointestinal adverse events attributable to semaglutide was performed according to the criteria outlined in the Naranjo Adverse Drug Reaction Probability Scale and the Common Terminology Criteria for Adverse Events (CTCAE, v5.0) (20, 21). Patients were classified into two groups: the incidence group, comprising those who experienced gastrointestinal adverse events, and the non-incidence group.

### 2.4 Statistical analysis

Statistical analyses were performed using SPSS 25.0, with a significance threshold set at *P* < 0.05. Categorical variables were presented as frequencies and percentages and analyzed using the chi-square test. Continuous variables with a normal distribution were expressed as means **±** standard deviations and compared using the *t*-test. Univariate and multivariate logistic regression analyses were employed to identify risk factors associated with gastrointestinal adverse events related to semaglutide use in patients with T2DM. Independent risk factors identified were subsequently incorporated into the R software environment (version 3.6.3), utilizing the rms package to develop nomograms predicting the likelihood of gastrointestinal adverse events in this patient population. The discriminative ability of the nomograms was assessed through receiver operating characteristic (ROC) curve analysis, while their calibration and goodness-of-fit were evaluated using calibration plots and the Hosmer–Lemeshow test, respectively.

## 3 Results

### 3.1 Gastrointestinal adverse events

Gastrointestinal adverse events attributable to semaglutide administration were observed in 41 of 215 patients with T2DM, representing an incidence rate of 19.07%. Some patients experienced multiple symptoms. Specifically, the reported events included abdominal pain in 15 patients, abdominal distension in 11, diarrhea in 9, nausea and vomiting in 9, and decreased appetite in 7. The gastrointestinal adverse events were graded according to CTCAE 5.0, with 21 cases classified as mild, 17 as moderate, and 3 as severe.

### 3.2 General information comparison

The incidence and non-incidence groups exhibited no statistically significant differences (*P* > 0.05) in terms of gender, age, body weight, duration of T2DM, glycosylated hemoglobin levels, BMI, ALT, AST, TCHO, TG, LDL-C, HDL-C, SBP, and DBP, smoking status, insulin, numbers of oral medication, concurrent use of metformin, sulfonylureas, thiazolidinediones, SGL2 inhibitors, DPP-4 inhibitors, and renal function status. Conversely, statistically significant differences were observed between the groups regarding the presence of concomitant gastrointestinal disease, alcohol consumption, and α-glycosidase inhibitors (*P* < 0.05, **Table 1**).

**Table 1:** General information.

Index	Incidence group	Non-incidence group	χ²/*t*	*P-*value
Sex			2.671	0.102
Male	14 (34.15)	84 (48.28)	/	/
Female	27 (65.85)	90 (51.72)	/	/
Age (year)	56.72 ± 13.58	55.27 ± 12.04	0.677	0.499
Body weight (Kg)	80.97 ± 12.78	82.88 ± 13.21	0.838	0.403
T2DM duration (year)	10.63 ± 3.52	9.85 ± 2.93	1.473	0.142
Glycated hemoglobin (%)	9.25 ± 1.66	9.18 ± 2.72	0.158	0.875
BMI (Kg/m²)	28.48 ± 3.83	29.26 ± 3.14	1.370	0.172
ALT (U/L)	32.28 ± 10.71	34.07 ± 11.24	0.925	0.356
AST (U/L)	24.94 ± 8.23	24.48 ± 8.11	0.326	0.745
TCHO (mmol/L)	4.31 ± 1.13	4.62 ± 1.22	1.484	0.139
TG (mmol/L)	2.05 ± 0.67	2.16 ± 0.71	0.902	0.368
LDL-C (mmol/L)	2.71 ± 0.89	2.64 ± 0.87	0.461	0.645
HDL-C (mmol/L)	1.19 ± 0.28	1.15 ± 0.33	0.717	0.474
SBP (mmHg)	133.54 ± 13.48	131.35 ± 14.96	0.862	0.390
DBP (mmHg)	81.31 ± 9.15	80.04 ± 9.48	0.777	0.438
Gastrointestinal diseases			12.085	0.001
Yes	26 (63.41)	59 (33.91)		
No	15 (36.59)	115 (66.09)		
Alcohol			8.088	0.004
Yes	15 (36.59)	29 (16.67)		
No	26 (63.41)	145 (83.33)		
Smoking			2.515	0.113
Yes	3 (7.32)	30 (17.24)		
No	38 (92.68)	144 (82.76)		
Insulin			3.053	0.081
Yes	12 (29.27)	30 (17.24)		
No	29 (70.73)	144 (82.76)		
Oral medications (*n*)	2.74 ± 0.91	2.67 ± 0.88	0.455	0.649
Metformin			1.065	0.302
Yes	30 (73.17)	140 (80.46)		
No	11 (26.83)	34 (19.54)		
Sulfonylureas			2.247	0.134
Yes	4 (9.76)	7 (4.02)		
No	37 (90.24)	167 (95.98)		
α-glucosidase inhibitors			7.100	0.008
Yes	19 (46.34)	44 (25.29)		
No	22 (53.66)	130 (74.71)		
Thiazolidinediones			2.977	0.084
Yes	4 (9.76)	6 (3.45)		
No	37 (90.24)	168 (96.55)		
SGLT2 inhibitors			1.045	0.307
Yes	5 (12.20)	33 (18.97)		
No	36 (87.80)	141 (81.03)		
DPP-4 inhibitors			0.814	0.367
Yes	2 (4.88)	4 (2.30)		
No	39 (95.12)	170 (97.70)		
Renal function			1.120	0.290
Normal	10 (24.39)	30 (17.24)		
Abnormal	31 (75.61)	144 (82.76)		

P<0.05.

### 3.3 Multifactorial analysis

Logistic regression analysis identified concomitant gastrointestinal diseases [95% confidence interval (CI): 2.074–9.808, *P* < 0.001], alcohol consumption (95% CI: 1.304–6.633, *P* = 0.009), and coadministration of α-glucosidase inhibitors (95% CI: 1.368–6.460, *P* = 0.006) as independent risk factors for gastrointestinal adverse events in patients with T2DM treated with semaglutide (**Table 2**). The predictive model for gastrointestinal adverse events is represented by the following logistic regression equation: Logit (*P*) = 1.506 × (presence of concomitant gastrointestinal disorders; coded as 0 = No, 1 = Yes) + 1.079 × (alcohol consumption; 0 = No, 1 = Yes) + 1.089 × (coadministration of α-glucosidase inhibitors; 0 = No, 1 = Yes) − 2.849.

**Table 2:** Multi-factor analysis.

Factor	Gastrointestinal disease	Alcohol	α-glucosidase inhibitors	Macronutrient
Description of the assignment	"No" = 0, "Yes" = 1	"No" = 0, "Yes" = 1	"No" = 0, "Yes" = 1	/
Beta coefficient	1.506	1.079	1.089	-2.849
Standard error	0.396	0.415	0.396	0.389
Wald statistic	14.449	6.754	7.568	53.667
*P-*value	<0.001	0.009	0.006	<0.001
Odds ratio	4.511	2.941	2.973	0.058
95%CI	2.074–9.808	1.304–6.633	1.368–6.460	/

### 3.4 Development of a column-line graph model

**Figure 1:**
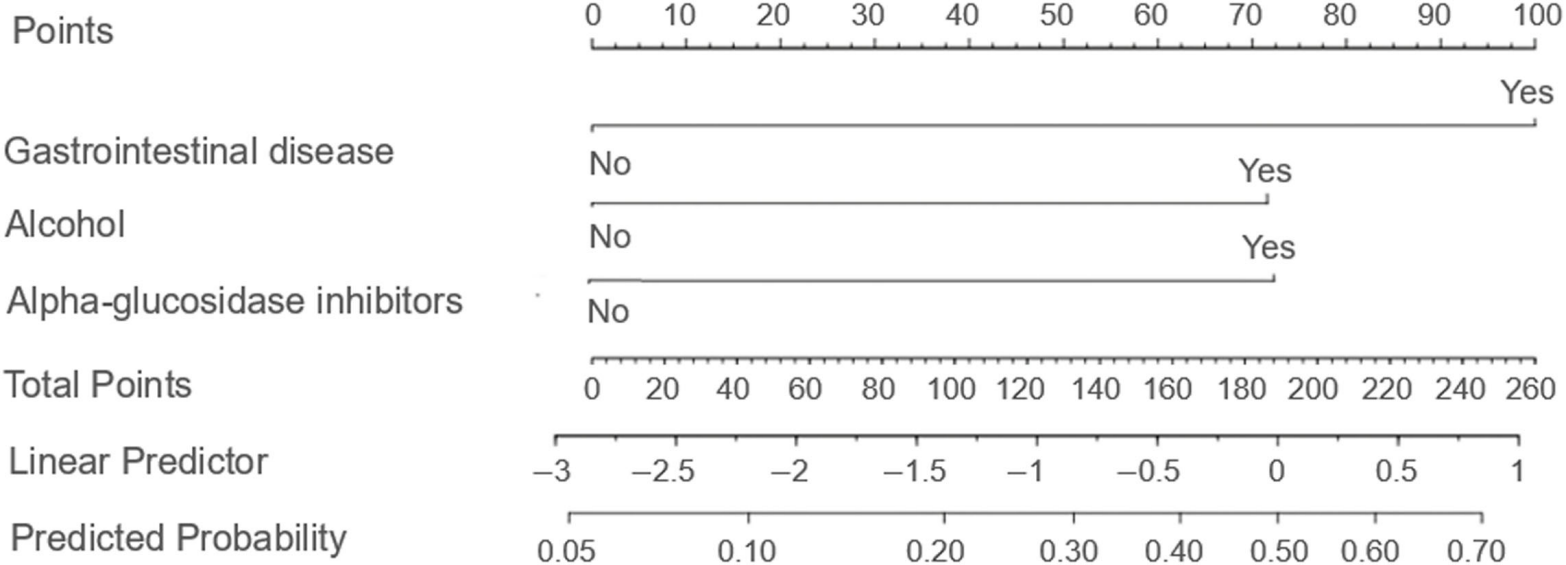
Column line diagram model. The nomogram incorporates three independent predictors determined through multivariate logistic regression analysis: gastrointestinal disease, alcohol consumption, and concurrent use of α-glucosidase inhibitors. Each predictor is allocated a specific point value on a graduated scale, with binary responses (e.g., "Yes" or "No") corresponding to distinct scores. The cumulative points are then mapped onto a risk probability scale ranging from 0% to 100% at the base of the nomogram, allowing clinicians to quantitatively assess individualized risk. This instrument facilitates rapid risk stratification and aids clinical decision making aimed at enhancing drug safety.

Based on the findings from the logistic regression analysis, three independent risk factors were identified: the presence of gastrointestinal diseases, alcohol consumption, and concurrent administration of α-glucosidase inhibitors. These variables were used to develop a column-line graph model designed to predict gastrointestinal adverse events associated with semaglutide use in patients with T2DM (**Figure 1**; **Table 3**).

**Table 3:** Scores for the column-line diagram model.

Predictor variable		Score (points)
Gastrointestinal disease	No	0
	Yes	100.0
Alcohol	No	0
	Yes	72.1
α-glucosidase inhibitors	No	0
	Yes	72.4

### 3.5 Evaluation of the column-line diagram model

**Figure 2:**
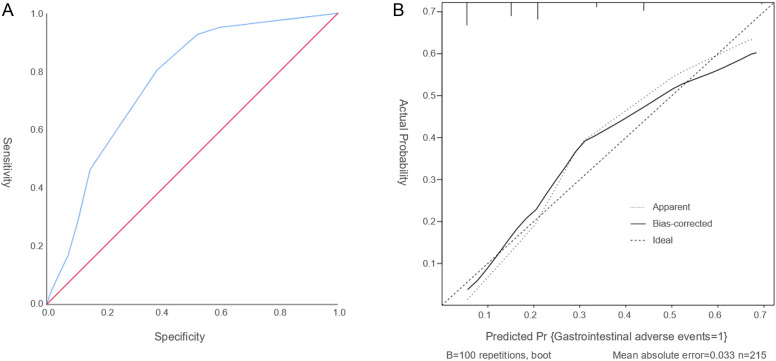
The ROC curve and calibration curve. **(A)** The ROC curve serves as a tool to assess the discriminatory capacity of the predictive model. **(B)** The Calibration curve evaluates the accuracy of the predicted probabilities. The calibration plot reveals a slope close to 1, signifying a strong concordance between the predicted probabilities and the observed incidence rates.

ROC curve is used to assess the discriminatory ability of the predictive model. The model’s area under the curve (AUC) is 0.763 (95% CI: 0.691–0.836, **Figure 2A**), reflecting a moderate to good level of discrimination. It indicates that the model reliably distinguishes between patients at risk of experiencing gastrointestinal adverse events induced by semaglutide and those who are not, demonstrating a balanced trade-off between sensitivity and specificity.

The calibration curve evaluates the accuracy of the predicted probabilities. The calibration plot reveals a slope close to 1, indicating a strong concordance between the predicted probabilities and the observed incidence rates (**Figure 2B**). Furthermore, the Hosmer–Lemeshow goodness-of-fit test yielded a chi-square statistic of 5.633 with a *P*-value of 0.228, suggesting an acceptable model fit. Together, these results confirm that the model provides reliable and precise risk predictions for gastrointestinal adverse events associated with semaglutide treatment in patients with T2DM. Overall, these evaluations support the robustness and reliability of the nomogram in forecasting the likelihood of semaglutide-related gastrointestinal adverse events.

## 4 Discussion

A significant proportion of patients with T2DM do not exhibit typical clinical symptoms during the early stages of the disease. However, without timely management of hyperglycemia and associated metabolic disturbances, these patients are at substantial risk of developing both acute and chronic complications (22). Since the introduction of GLP-1 receptor agonists (GLP-1 RAs) for T2DM treatment, their utilization has increased markedly among this patient population. Given the large number of individuals receiving GLP-1 RAs, gastrointestinal adverse events related to these agents have been frequently reported, leading to decreased medication adherence and adversely affecting therapeutic outcomes (8, 23, 24). Considering the relatively recent market approval of semaglutide, comprehensive safety data remain limited, necessitating further rigorous investigations to establish a robust evidence base for its safe clinical application. In the present study, 41 of 215 T2DM patients (19.07%) experienced gastrointestinal adverse events attributed to semaglutide administration. As a GLP-1 receptor agonist, semaglutide inhibits peristalsis in the gastric antrum and relaxes the gastric fundus, thereby significantly slowing the rate at which gastric contents are discharged into the duodenum. Additionally, semaglutide acts on GLP-1 receptors in the hypothalamus to produce an appetite-suppressing effect, which may underlie the gastrointestinal adverse events associated with its use (12). This finding indicates a relatively high incidence of gastrointestinal side effects associated with semaglutide in this patient cohort, underscoring the importance of vigilant monitoring and proactive strategies to mitigate these adverse events during treatment.

This study investigated the determinants influencing the incidence of gastrointestinal adverse events associated with semaglutide administration in patients with T2DM. The objective was to facilitate the proactive identification of appropriate interventions to mitigate such adverse events during clinical use. Logistic regression analysis identified three independent risk factors for gastrointestinal adverse events in this patient population. (I) The presence of concomitant gastrointestinal disorders. Although gastrointestinal disease is not a contraindication for semaglutide use, the drug’s effect in delaying gastric emptying may exacerbate symptoms in T2DM patients with existing gastrointestinal conditions (25). Consequently, cautious administration is recommended for patients with severe gastrointestinal disorders (e.g., gastroparesis), starting treatment at a low dose and closely monitoring for any gastrointestinal discomfort. (II) Alcohol consumption. Chronic and excessive alcohol intake can compromise the integrity of the gastric mucosa, increasing susceptibility to gastrointestinal discomfort (26). Therefore, enhanced patient education is warranted to promote healthy lifestyle behaviors and strict limitation of alcohol consumption among T2DM patients. (III) Concurrent use of α-glucosidase inhibitors. These inhibitors affect carbohydrate absorption in the small intestine, leading to fermentation of unabsorbed carbohydrates by colonic bacteria, which can cause abdominal distension and increased gas (27). That is to say, before prescribing semaglutide, doctors should conduct a thorough assessment of the patient’s digestive tract conditions, drinking habits, and current medications. For patients with risk factors, it is recommended to start with a low dose and gradually increase it while informing patients about the potential side effects.

Additionally, a nomogram was developed incorporating concomitant gastrointestinal diseases, alcohol consumption, and the co-administration of α-glucosidase inhibitors to assess its effectiveness in predicting gastrointestinal adverse events associated with semaglutide use in patients with T2DM. The predictive performance of the nomogram was evaluated using calibration curve analysis, which demonstrated a slope close to 1, alongside the Hosmer–Lemeshow goodness-of-fit test. These assessments indicated that the nomogram’s predictions closely corresponded to the observed incidence of gastrointestinal adverse events in this patient population. Furthermore, the discriminative ability of the model was supported by an area under the ROC curve of 0.763, suggesting satisfactory differentiation. Numerous international studies have examined gastrointestinal adverse events associated with GLP-1 RAs. For example, the multinational SUSTAIN clinical trial program evaluating semaglutide reported gastrointestinal adverse events—such as nausea, vomiting, and diarrhea—in approximately 10.5%–21.9% of participants, a prevalence closely comparable to the 19.07% observed in the present study (28). Moreover, a retrospective study conducted in the United States demonstrated that patients with a prior history of gastrointestinal disorders exhibited a significantly elevated risk of gastrointestinal adverse events when treated with GLP-1 RAs, corroborating the current study’s identification of concomitant gastrointestinal diseases as an independent risk factor (29). A recent study further showed that adverse events occurring during treatment, as well as treatment discontinuations attributable to adverse events—predominantly gastrointestinal in nature—were more frequent in the semaglutide 8 mg and 16 mg dosage groups compared to the 2 mg group among individuals with type 2 diabetes and overweight or obesity (30). Notably, international research has largely overlooked the influence of alcohol consumption and the concurrent use of α-glucosidase inhibitors on gastrointestinal adverse events, indicating that the findings reported here may introduce novel risk factors relevant to clinical management.

The nomogram model developed in this study holds significant clinical relevance. First, it enables personalized risk assessment by quantifying the impact of concomitant gastrointestinal disorders, alcohol consumption, and the concurrent administration of α-glucosidase inhibitors. This allows clinicians to more accurately estimate the likelihood of patients experiencing gastrointestinal adverse events, thereby facilitating the development of individualized treatment regimens. Second, the model guides optimized medication strategies; for patients identified as high-risk—such as those with multiple risk factors—initiating semaglutide therapy at a reduced dosage combined with intensified monitoring may enhance treatment safety and improve patient adherence. Third, this research addresses existing gaps in the literature, as predictive models for semaglutide-related gastrointestinal adverse events remain scarce both domestically and internationally. The present study offers a foundational tool in this area, with future multicenter validation studies expected to improve the model’s applicability and generalizability. However, the existing model is based solely on data related to semaglutide. Since semaglutide has distinct pharmacokinetic properties and receptor affinity compared to other GLP-1 receptor agonists (such as liraglutide and dulaglutide), the applicability of this model to predict outcomes for other GLP-1 receptor agonists requires further validation. It should also be pointed out that semaglutide has also been used for the treatment of weight loss, with gastrointestinal adverse events commonly occurring during therapy (31, 32). Therefore, whether our current model is applicable for predicting the effects of GLP-1 receptor agonists on weight loss still requires further investigation.

This study has several limitations. First, “drink” is only defined as the regular intake of any alcoholic beverage for more than six months. However, daily alcohol consumption was not further classified into different levels in our present study. Second, it is a single-center investigation involving a relatively small cohort of 215 patients with T2DM sourced from the same hospital. Notably, regional differences in patients’ lifestyles, medication habits, ethnic composition, and the quality of diagnosis and treatment may affect the generalizability of the model. Third, the current column chart includes only three predictive variables and does not encompass all significant influencing factors. Additional variables, such as dietary patterns, psychological status including depression, anxiety, and treatment compliance, as well as gut microbiota, may also affect the accuracy of long-term predictions. Fourth, the absence of external validation limits the assessment of the generalizability of the developed column chart. Future research will adopt a multicenter design to increase the sample size and diversify the patient population, thereby enabling more robust validation of potential independent risk factors for gastrointestinal adverse events associated with semaglutide use in patients with T2DM. Additionally, external validation will be conducted following refinement of the column charts, and the development of dynamic column charts is planned to enhance their applicability in clinical practice.

## 5 Conclusion

The incidence of gastrointestinal adverse events associated with semaglutide use in patients with T2DM was found to be elevated. Independent risk factors identified included the presence of concurrent gastrointestinal disorders, alcohol consumption, and the co-administration of α-glucosidase inhibitors. Furthermore, the developed nomogram may serve as a predictive tool for assessing the risk of gastrointestinal adverse events related to semaglutide treatment in this patient population.

## Data Availability

The raw data supporting the conclusions of this article will be made available by the authors, without undue reservation.
